# A Novel Approach to Obtaining Metal Oxide HAR Nanostructures by Electrospinning and ALD

**DOI:** 10.3390/ma16237489

**Published:** 2023-12-03

**Authors:** Blagoy S. Blagoev, Borislava Georgieva, Kirilka Starbova, Nikolay Starbov, Ivalina Avramova, Krastyo Buchkov, Peter Tzvetkov, Rumen Stoykov, Penka Terziyska, Damyan Delibaltov, Vladimir Mehandzhiev, Albena Paskaleva

**Affiliations:** 1 Institute of Solid State Physics, Bulgarian Academy of Sciences, 72 Tsarigradsko Chaussee, 1784 Sofia, Bulgaria; b.georgiewa@abv.bg (B.G.); ilka_05@yahoo.com (K.S.); kikostar@mail.bg (N.S.); k.buchkov@hotmail.com (K.B.); penka@issp.bas.bg (P.T.); damyandelibaltov@gmail.com (D.D.); vlado_bm@yahoo.com (V.M.); paskaleva@issp.bas.bg (A.P.); 2 Institute of Electronics, Bulgarian Academy of Sciences, 72 Tsarigradsko Chaussee, 1784 Sofia, Bulgaria; 3 Institute of General and Inorganic Chemistry, Bulgarian Academy of Sciences, 1113 Sofia, Bulgaria; iva@svr.igic.bas.bg (I.A.); tzvetkov@svr.igic.bas.bg (P.T.); 4 Central Laboratory of Solar Energy and New Energy Sources, Bulgarian Academy of Sciences, 1784 Sofia, Bulgaria; rstoykov@gmail.com

**Keywords:** electrospinning, atomic layer deposition (ALD), high-aspect-ratio (HAR) nanostructures, ZnO, polyvinyl alcohol (PVA)

## Abstract

In this work, a novel approach is suggested to grow bilayer fibers by combining electrospinning and atomic layer deposition (ALD). Polyvinyl alcohol (PVA) fibers are obtained by electrospinning and subsequently covered with thin Al_2_O_3_ deposited at a low temperature by ALD. To burn the PVA core, the fibrous structures are subjected to high-temperature annealing. Differential scanning calorimetry (DSC) analysis of the PVA mat is performed to establish the proper annealing regime for burning off the PVA core and obtaining hollow fibers. The hollow fibers thus formed are covered with a ZnO layer deposited by ALD at a higher temperature within the ALD window of ZnO. This procedure allows us to prepare ZnO films with better crystallinity and stoichiometry. Different characterization methods—SEM, ellipsometry, XRD, and XPS—are performed at each step to investigate the processes in detail.

## 1. Introduction

Recent decades have seen a huge increase in the world’s human population that has affected the global economy, politics, and industry. To solve some of these problems worldwide, great efforts are being made in the development of nanotechnologies. They provide opportunities for producing materials and structures with unique properties that can be tailored to satisfy the requirements of specific applications, e.g., for low energy consumption and high efficiency, which is key in addressing the growing environmental problems. One of the reasons why nanomaterials have very interesting and unique properties (as compared to their bulk counterparts) is that they have a huge surface area compared to their volume. Furthermore, by reducing the size of an object to its structural units (atoms and molecules), the properties of materials may be substantially changed, as they are then governed by quantum laws. A large number of advanced techniques have been proposed with the purpose of producing nanomaterials. Here, we are focused on two of them: electrospinning and atomic layer deposition.

Electrospinning is a low-cost fiber production method that uses high electrostatic voltage to draw charged filaments from polymer solutions to fiber diameters ranging from tens of nanometers to tens of micrometers [1,2,3,4]. Using this method, different structures are obtainable depending on the experimental parameters and conditions: beads, fibers with beads, polymeric, inorganic, and composite fibers, either solid or hollow [3,5]. The permanent and growing interest in the electrospinning process since its patenting in 1943 by Anton Formhals [6] has been due to it allowing easy preparation of various high-aspect-ratio (HAR) structures that find a wide variety of applications: filtration systems, catalysis, energy harvesting, biomedical engineering, flexible devices, batteries, sensors, water purification, etc. [2,4,7,8,9]. One of the most widely used electrospinning polymers is polyvinyl alcohol (PVA) because it is easy to work with and is environmentally friendly [10,11].

Atomic layer deposition (ALD) is a vapor-phase technique that produces thin films of a variety of materials. Based on sequential self-limiting surface reactions, ALD offers excellent conformality on high-aspect-ratio (HAR) structures, as well as step coverage, precise thickness control at an angstrom level, continuous pinhole-free layers, and tunable film composition [12,13]. Still, another advantage of the ALD technique is the relatively low deposition temperature. These advantages formed the basis of many interesting applications that are impossible or quite difficult to achieve by other thin-film deposition methods. Thus, the ALD has found a large number of industrial applications in microelectronics, magnetic heads, thin film electroluminescent (TFEL) displays, protective coatings, optics, coatings on powders, MEMS devices, energy storage and conversion, desalination, catalysis, and medicine [14,15].

Combining electrospinning and ALD is a powerful and flexible approach [16] to revealing the advantages of both techniques and creating smart devices for various green technology applications. This innovative technique for preparing HAR structures opens up a large number of applications: (photo)catalysis, solar cells, sensors, batteries, photodetectors, devices for organic pollutant removal, bioactive coatings, and fibers for bone implants and scaffolds [16,17,18,19,20,21,22,23,24,25]. To produce hollow coaxial two- or multilayer fiber structures, the following sequence is usually followed: electrospinning of polymer fibers, low-temperature ALD of the desired films in one ALD process, and annealing the structure at a sufficiently high temperature to burn off the polymeric core. During annealing, the Kirkendall effect takes place due to the different diffusion coefficients of adjacent films, which consists of a motion of the interface between two metals arising as a consequence of the difference in the metal atoms’ diffusion rates. For example, a significant disruption of the microtubular morphology was observed during the annealing of ZnO/Al_2_O_3_ (ZnO/ALO) structures obtained on a polyvinyl alcohol (PVA) mat template [26].

In this work, we combined electrospinning and ALD in a novel two-step approach to fabricate hollow ZnO/ALO bi-layer submicron fibers on a glass substrate. In the first step, submicron PVA fibers were coated with an ALO film by using low-temperature ALD. The ALO films obtained by ALD are amorphous and can be deposited at a very low temperature (even at 50 °C) with a relatively good quality. The ALO film serves as a scaffold because it is thermally and chemically stable. This was followed by high-temperature annealing to burn off the polymer. In the next step, ZnO was deposited by thermal ALD at temperatures within the range of its ALD window. This two-step ALD process (low-temperature and conventional thermal ALD) provides a better quality of the topmost ZnO film by preventing the Kirkendall effect from taking place. To find a suitable annealing temperature regime, a DSC analysis of the PVA mat was performed beforehand. SEM, ellipsometry, XRD, and XPS analyses were conducted at each process step.

## 2. Materials and Methods

A series of samples were obtained, and their physical and chemical properties were investigated by various methods following the deposition and measurement steps given below:

- Cleaning the glass and Si substrates;
- Electrospinning of PVA polymer on glass substrates;
- Differential scanning calorimetry (DSC) of the PVA mat;
- Scanning electron microscopy (SEM) of the PVA polymer on glass substrates;
- Low-temperature ALD of Al_2_O_3_ (ALO) films on submicron electrospun PVA fibers; and on flat reference substrates (glass, Si);
- Ellipsometry, XRD, XPS, and SEM measurements;
- Thermal annealing of all samples to remove the PVA core and/or anneal the ALO film;
- Ellipsometry of the annealed ALO films;
- ALD of ZnO on the as-obtained hollow ALO submicron fibers and on referent flat substrates: glass, Si, ALO/glass, ALO/glass ann, ALO/Si, and ALO/Si ann;
- Ellipsometry, XRD, and XPS measurements.

**Figure 1:**
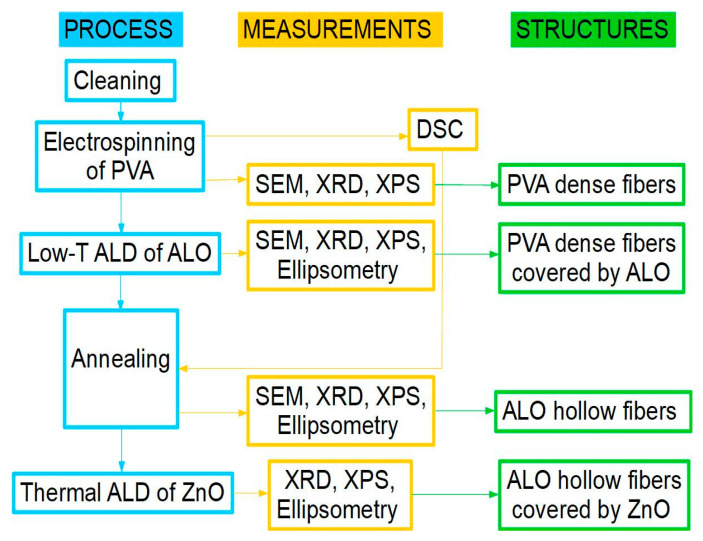
Block diagram of the experimental procedures.

The block diagram of the experimental procedures is presented in Figure 1.

### 2.1. Sample Preparation

The template fibers were synthesized by electrospinning of a 10 wt% aqueous solution of high-molecular-weight (7200 MW) polyvinyl alcohol (PVA, VALERUS, 93.5%). The solution was obtained at 60 °C under continuous stirring and had excellent viscoelastic behavior under electrospinning conditions. A syringe with a stainless-steel needle was filled with the solution and subjected to a field with a strength of 2.0 kV/cm in homemade electrospinning equipment at an ambient temperature of about 23 °C and a flow rate of 0.15 mL/hour. As a result, template PVA fibers having good adhesion to the surface of flat glass substrates were obtained.

The Al_2_O_3_ (ALO) films were prepared by ALD in a Beneq TFS-200 reactor. Trimethylaluminum (TMA) and deionized water (DI H_2_O) were sequentially introduced in the reactor as precursors under their own vapor pressures at 19 °C. Nitrogen was used as a purging gas, the pulse and purging times being the same for both precursors, 300 ms and 5 s, respectively. The ALD cycle was repeated 200 times. To protect the polymer from thermal degradation, the temperature was maintained at 60 °C. After the ALO deposition, the samples were annealed in air in a Carbolite horizontal tube furnace equipped with a Eurotherm 3508 thermocontroller. The thermal regime consisted of three steps: (i) increasing the temperature up to 500 °C at a 5 °C/min ramp rate; (ii) a 24-h-dwell period at 500 °C; and (iii) cooling down to room temperature at a rate of 5 °C/min. Further, ZnO was also deposited on the ALO films by again using ALD. Diethylzinc (DEZ) and DI H_2_O were the precursors in a deposition cycle repeated 155 times and consisting of 300-ms pulse duration separated by a 5-s purging by pure nitrogen gas at a temperature of 180 °C.

In each run, together with the PVA mat-covered substrates, a pure glass, and a Si substrate were placed in the reactor chamber to use as references.

### 2.2. Characterization Techniques

Differential scanning calorimetry (DSC) was performed by a NETZSCH DSC 200 PC device; the results were analyzed using the NETZSCH Proteus Thermal Analysis software, version 4.3.1. Two crucibles were placed side-by-side in the DSC apparatus chamber, one with the substance to be measured and an empty one as a reference sample. Holes were drilled on the crucible’s top to allow the gas to evaporate. Liquid nitrogen and its vapor were used to control the temperature and as a purging and protective gas. The gas flow rate was 20 mL/min. The measurement was carried out in the temperature range from room temperature to 550 °C and vice versa; the temperature was held at 550 °C for 15 min. The heating and cooling rates were 5 °C/min. The total mass of PVA material placed within the crucible was 15.410 mg; it decreased to 0.87 mg after the DSC measurement. The enthalpy was estimated from the area of the peak by tangential approximation.

A JSM T 200 (Jeol) scanning electron microscope (SEM) (Jeol, Akishima, Tokyo, Japan) was used for imaging and recording the fibers prepared. All fibers (as-spun, covered with ALO, and thermally processed) were non-conductive. This required that their imaging under an electron microscope be preceded by consecutive vacuum deposition of nano-thick carbon and gold films via sputtering and thermal evaporation, correspondingly.

The structural characterization of the deposited layers was carried out by X-ray diffraction (XRD) patterns using a Bruker D8 Advance diffractometer (Bruker AXS, Karlsruhe, Germany) equipped with a copper tube (CuKα_1,2_ radiation) and a LynxEye PSD detector (3.4° 2θ effective matrix area). The X-ray diffraction patterns were collected at room temperature in the angular range from 5.5 to 90.0° 2θ, step of 0.04° 2θ, and a total of 70 s/step integrated counting statistics. The qualitative phase analysis was performed by using the Bruker Diffrac.Eva 4.0 software and the ICDD PDF-2 (release 2021) database.

X-ray photoelectron spectroscopy was also applied for the investigation of the samples. These analyses were performed on a Kratos AXIS Supra spectrometer (Kratos Analytical Ltd., Manchester, UK) with an achromatic Al Kα radiation with an energy of 1486.6 eV under a vacuum better than 10^−8^ Pa at a 90-degree take-off angle. The accuracy of the measured BE was 0.1 eV. The spectrometer resolution was calculated from the Ag 3d5/2 line with an analyzer transmission energy of 20 eV. The spectrometer was calibrated against the Au 4f7/2 line (84.0 eV). Each analysis started with a survey scan from 0 to 1200 eV, with a pass energy of 160 eV at steps of 0.5 eV with two sweeps. For the high-resolution analysis, the number of sweeps was increased, and the pass energy was lowered to 20 eV at steps of 100 meV. Charge compensation was used during the measurements. The sample charging was estimated from the C 1s (285 eV) spectra of the natural hydrocarbon contaminations on the surface. The photoelectron lines of the constituent elements on the surface were recorded and corrected by subtracting the Shirley-type background and quantified using, the peak area and Scofield’s photoionization cross-sections. The spectra deconvolution was conducted by the XPSPEAK41 software.

The referent aluminum oxide (ALO) and zinc oxide (ZnO) film thicknesses deposited on flat glass and Si substrates were determined by spectroscopic ellipsometry using a Woollam M2000D ellipsometer (J.A. WOOLLAM CO. Inc., Lincoln, NE, USA). 

## 3. Results and Discussions

**Figure 2:**
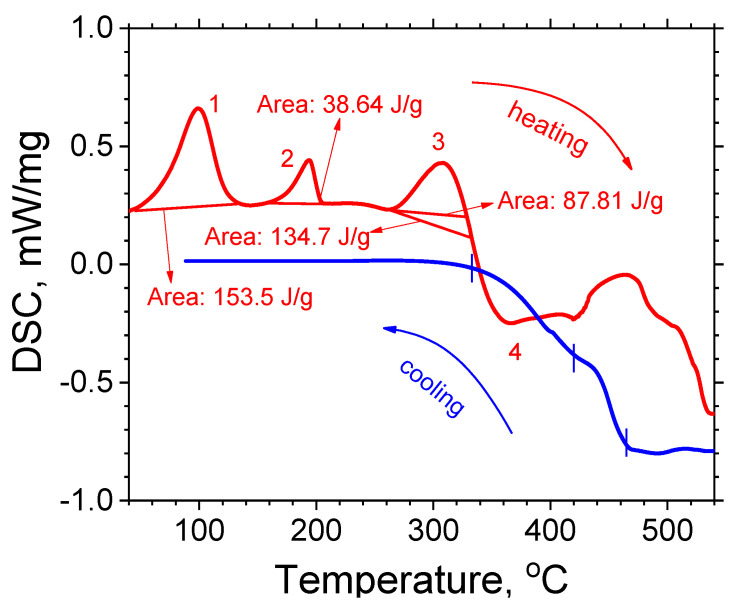
DSC thermogram of PVA.

The purpose of the DSC analysis performed was to analyze the PVA mat’s thermal characteristics and obtain the thermal annealing process parameters. The DSC thermogram (Figure 2) clearly indicates various complicated enthalpy changes in the PVA. During the heating process, three endothermic reactions are observed, followed by one exothermic reaction. During the cooling, the exothermic reaction continues down to 333 °C. 

The first endothermic process (peak 1 in Figure 2) starts at 69.8 °C, reaches a maximum at 99.0 °C and ends at 121.9 °C. The enthalpy value of this peak is 153.5 J/g. Based on the temperature range, the width, and the shape of the peak, one can assume that it arises due to the material’s drying. The second endothermic process (peak 2 in Figure 2) starts at 175.6 °C, has a maximum at 193.9 °C, and ends at 203.5 °C; its enthalpy value is 38.64 J/g. The thermogram reveals that the amount of heat required to raise the temperature before and after the second peak is approximately the same. Therefore, one can conclude that this peak results from a single first-order phase transition. The third endothermic process (peak 3 in Figure 2) starts at 271.2 °C, has a maximum at 307.7 °C, and ends at 328 °C, with an enthalpy value of 87.81 J/g. This peak is related to the beginning of a thermal decomposition process. The sample continues to absorb heat up to 333 °C, where the enthalpy increases up to 134.7 J/g. An inflection point at 333 °C is seen—above this temperature, the process is irreversible, and a complex exothermic reaction begins (valley 4 in Figure 2). Above 440 °C, the curve behavior is more complicated, which implies that several chemical reactions take place simultaneously. The exothermic reaction continues during the cooling process, regardless of the temperature drop. In the exothermic process, the sample’s enthalpy change is negative. Simultaneously, the mass of the sample decreases, i.e., gases are released, and, therefore, the entropy change is positive. The change in Gibbs energy, dG, can be obtained from Equation (1):

dG = dH − TdS,(1)

where H is the enthalpy, T is the absolute temperature, and S is the entropy. Therefore, the change in Gibbs energy is negative, which is representative of an irreversible chemical reaction. In the cooling process, a multi-stage oxidation reaction probably takes place (see the marks in the cooling process in Figure 2). Usually, for an irreversible chemical reaction to start, a certain energy barrier has to be overcome. The activation energy is the minimum amount of energy that has to be provided to compounds to cause a chemical reaction. The third endothermic process provides 134.7 J/g of energy to initiate the reaction considered. The temperature of 333 °C is the inflection point of the thermogram, after which the oxidation process is self-sustaining.

The thermal characteristics and the fact that almost the entire PVA amount was evaporated during the thermal treatment gave us reason to use a similar regime for heating the PVA but with keeping it for 24 h, as in [27]. Thus, annealing at 500 °C for 24 h was used to remove the PVA core of the fibers.

The spectroscopic ellipsometry analyses were performed on (i) ALO films deposited on Si and glass substrates, on (ii) ZnO films deposited on Si and glass substrates, and on (iii) the ZnO films from (ii) deposited on the ALO substrates from (i). The experimental Ψ and Δ were analyzed using a multi-layer model consisting of:

(i) a silicon substrate with a 2.5-nm silicon native oxide as a first layer, an ALO layer as a second layer, and a roughness layer;
(ii) a silicon substrate with a 2.5-nm silicon native oxide, a ZnO layer as a second layer, and a roughness layer;
(iii) a silicon substrate with a 2.5-nm silicon native oxide, an ALO layer with its parameters determined in (i), a ZnO layer, and a roughness layer.

For the Si substrate and the native oxide, the data used was taken from the database of the CompleteEASE v.5.19 (J.A.Woollam Co. Inc, Lincoln, NE, U.S.) data analysis software. The ALO layer was represented by a Cauchy dispersion. The ZnO layer was modeled using a PSemi-M0 and one Gaussian oscillator. The roughness layer for all samples was modeled by using Bruggeman’s EMA (Effective Medium Approximation) of 50% voids and 50% bulk material.

**Figure 3:**
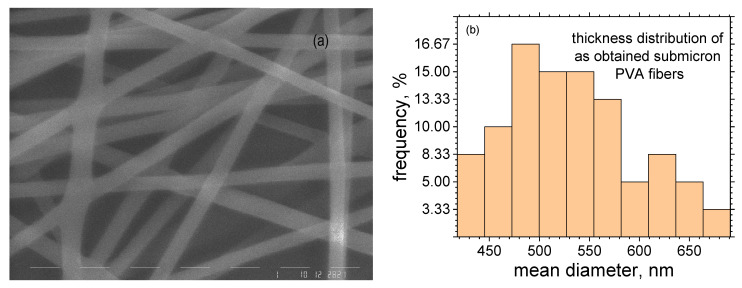
SEM image (**a**) and thickness distribution of obtained submicron PVA fibers (**b**).

The obtained electrospun PVA fibers were well-formed and almost endless (Figure 3a). Their mean diameter was 534.11 nm, with a standard deviation of ~66.94 nm obtained by 60 measurements along the fiber length. The diameter distribution is presented in Figure 3b.

**Figure 4:**
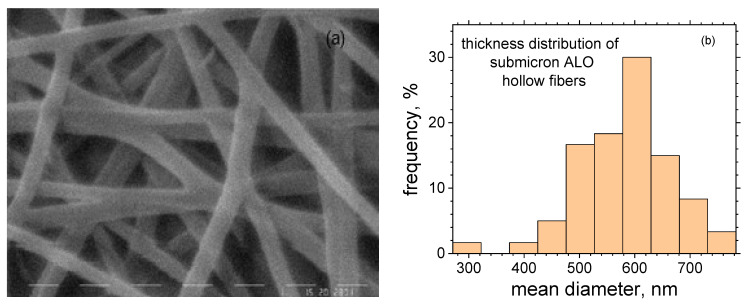
SEM image (**a**) and thickness distribution of submicron ALO hollow fibers (**b**).

By covering the submicron PVA fibers with Al_2_O_3_ (ALO) film and subsequent annealing, their structure was transformed from that of solid polymer fibers to hollow ALO fibers with a preserved geometry (Figure 4a). During the annealing, the PVA polymer was burned off, as proven by the DSC analysis (Figure 2). The mean thickness diameter increased to 579.89 nm with a standard deviation of 89.52 nm. The thickness distribution is presented in Figure 4b.

The difference in the thicknesses of the as-spun fibers and those covered with ALO film was about 46 nm, which yields 23 nm for the ALO film thickness. This value is smaller than the mean thickness value measured by ellipsometry of the referent flat ALO films on Si and glass substrates, namely, 34.24 nm. This difference could be attributed to the PVA fibers shrinking during the ALD and annealing processes. It should also be noted that the initial growth rate is different for different surfaces. Furthermore, on HAR structures, in contrast to standard flat substrates, the desired film grows more slowly.

Further, ZnO films were deposited on the ALO fibers. The thickness of ZnO was about 170 nm, as measured by ellipsometry. The final result was ALO/ZnO hollow fibers with an inner diameter (cavity) of 512 nm and a 23-nm thick ALO layer covered by 170 nm of ZnO.

**Figure 5:**
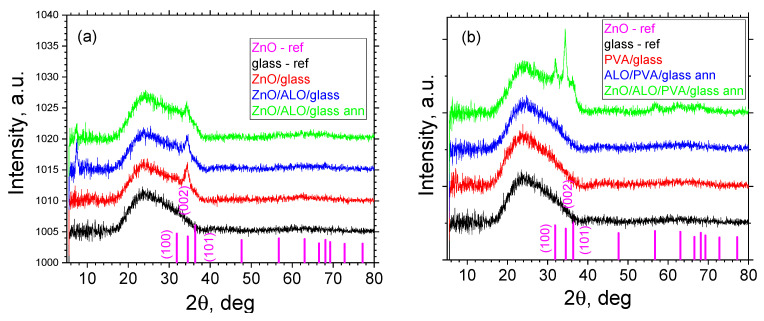
XRD reflexes of ZnO and ZnO/ALO structures on flat (**a**) and on fiber HAR (**b**) templates, all on glass substrates.

**Figure 6:**
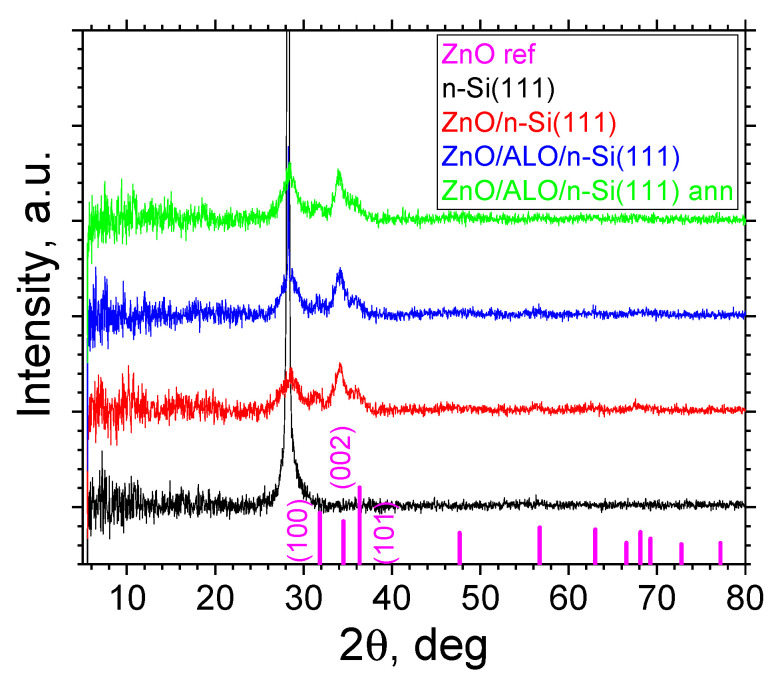
XRD reflexes of ZnO and ZnO/ALO structures on flat Si substrates.

XRD analysis was performed for the ZnO and ZnO/ALO structures deposited on the reference flat substrates (glass and n-Si(111)) and on fiber HAR templates on glass substrates (Figure 5 and Figure 6).

The resulting polyvinyl alcohol (PVA) fibers and the as-deposited aluminum oxide (ALO) film exhibit a diffraction pattern of an amorphous material, as seen in the data presented in Figure 5. In contrast, for the on-glass-deposited ZnO layers, the ZnO/ALO, and as-annealed ZnO/ALO layers, nanoscale polycrystalline ZnO layers are observed (Figure 5a). In all samples containing ZnO, only one reflection from the phase with index (002) is observed. An exception is the ZnO/ALO/PVA-annealed sample, where, in addition to the (002) peak, reflections with indices (100) and (101) are also visible. It is noteworthy that their intensity ratio differs significantly from the reference data, with the intensity of the (002) reflex being significantly higher than the other. The reason for this is the ZnO crystallites’ preferred orientation during the formation of the layer, as they are oriented along the crystallographic direction [00l] relative to the substrate. This is probably true for all samples where ZnO is observed as a crystalline phase. Most crystal orientations visible for ZnO on submicron fibers can be attributed to the curved surface (of the fibers) on which this film is grown. Additionally, shrinking during the heating processes also changes the ALO surface/structure, and the subsequent ZnO film grows differently on this modified surface.

The ZnO and ZnO/ALO layers deposited on an n-Si(111) substrate (Figure 6) show similar diffraction patterns to those just described. For them, one diffuse reflex of nanosized ZnO with index (002) is observed in all samples. In some structures, a small peak at (100) can also be observed. Obviously, the ZnO crystallites’ preferred orientation along the [00l] direction depends weakly on the substrate used. In addition, the annealing procedure does not significantly change the ZnO films’ crystal structure.

**Figure 7:**
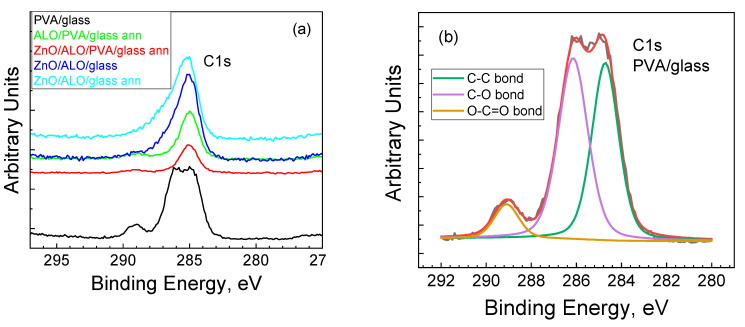
Binding energy of C1s spectra of PVA, ALO, and ZnO-containing structures deposited on glass substrates (**a**) and deconvolution spectra of PVA fibers (**b**).

XPS was used to study the surface composition of the different structures containing PVA, ALO, and ZnO deposited on glass substrates. The C 1s, Al 2p, Zn 2p, and O 1s photoelectron lines were recorded after each step of deposition of additional layers on top of the glass substrate. The first structures investigated were PVA fibers on a glass substrate (PVA/glass)—its characteristic bonds appearing in the C1s spectra are evident (Figure 7).

The C-C [28], C-O [29], and O-C=O [30] bonds are clearly distinguishable in the C1s spectra at 285 eV, 286 eV, and 289 eV, respectively (Figure 7b). In contrast, the other structures possess only C-C peaks at 285 eV (Figure 7a). It should be mentioned that the top layer with the least thickness, 23 nm, is that of ALO on the fibrous structure, while the depth of XPS information is about 10 nm. This means that only the C-C peak due to surface contamination can be visible. Nevertheless, the PVA must have burned off during the annealing, as evidenced by the DSC analysis.

**Figure 8:**
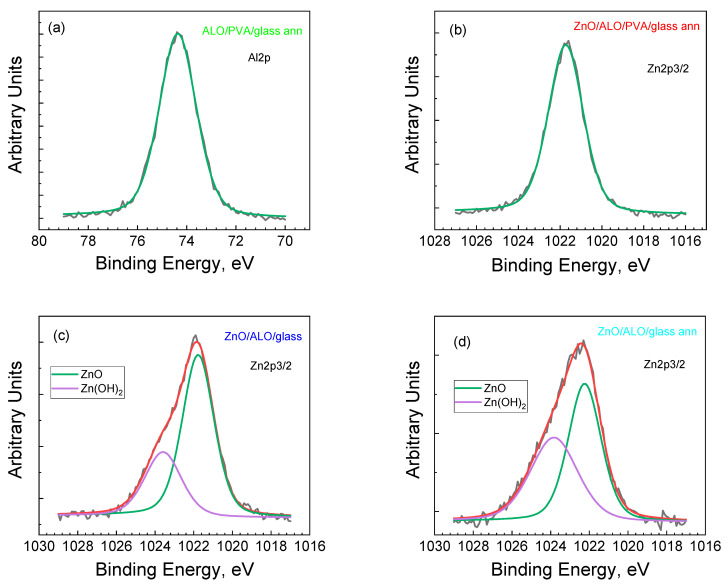
Deconvolution spectra of Al2p of ALO/PVA/glass ann (**a**); Zn2p3/2 of ZnO/ALO/PVA/glass ann (**b**); ZnO/ALO/glass (**c**); and ZnO/ALO/glass ann (**d**) structures.

**Figure 9:**
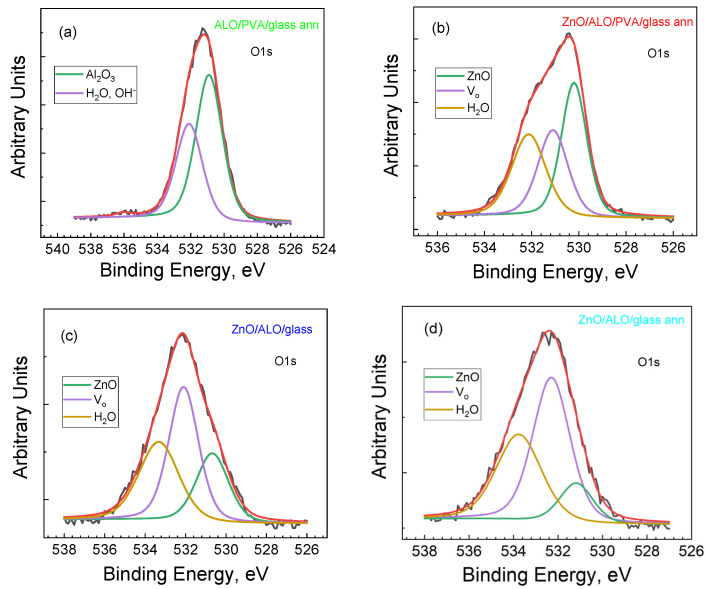
Deconvolution O1s spectra of ALO/PVA/glass ann (**a**); ZnO/ALO/PVA/glass ann (**b**); ZnO/ALO/glass (**c**); and ZnO/ALO/glass ann (**d**) structures, where Vo denote oxygen vacancies.

The XPS spectra of Al2p, Zn2p3/2, and O1s clearly prove the formation of ALO and ZnO films on fiber structures and on flat reference substrates (Figure 8 and Figure 9).

After covering the PVA fibers with ALO on a glass substrate and the subsequent annealing procedure (ALO/PVA/glass ann), the binding energy of Al2p is situated at 74.3 eV (Figure 8a), and the binding energy of O1s appears at 531.2 eV (Figure 9a), which is associated with the formation of Al_2_O_3_ oxide. The formation of ZnO is also proven over the as-prepared ALO/PVA/glass ann structure. The recoded spectra of Zn 2p and O 1s confirm this (Figure 8b and Figure 9b). The Zn 2p photoelectron spectrum shows a shape and binding energies positions typical for ZnO (Figure 8b). In the O 1s spectra, there are three peaks (Figure 9b–d) associated with the Zn-O bond in a ZnO wurtzite structure (~530 eV), oxygen associated with the formation of vacancies in the ZnO lattice (~531 eV), and other types of adsorbed oxygen (~532 eV), such as H_2_O [31,32].

The Zn 2p3/2 and O 1s spectra of ZnO/ALO/glass and ZnO/ALO/glass ann flat structures (Figure 8c,d and Figure 9c,d) show that in addition to ZnO, the formation of Zn(OH)_2_ occurs, which is demonstrated by the second peak at a higher binding energy of around 1024 eV in Zn 2p3/2. Moreover, in the O 1s spectra, the peaks corresponding to oxygen vacancy and adsorbed oxides are shifted to the higher energies, with the vacancy peak being the highest energy component. The annealing does not create significant changes, except for decreasing the absorbed oxygen and the small shift to the higher energies of all O 1s peaks. The oxygen mobility in flat structures is higher, and, therefore, more vacancies are present in them, which is responsible for their higher conductivity.

Both Zn 2p and O 1s spectra imply the formation of non-stoichiometric ZnO on flat substrates. On the contrary, the films deposited on a fibrous structure exhibit a substantially improved stoichiometry, demonstrated by the absence of minor peaks in the Zn 2p spectrum and the substantial reduction of the 531 eV and 532 eV peaks in the O 1s spectrum. Therefore, the XPS reveals that the ZnO layers deposited on fibrous structures are of a better quality compared to the layers deposited on flat substrates.

It is helpful to compare our technology of preparing hollow fibers with those suggested by other authors. So far, the combined electrospinning/ALD methods of obtaining bilayer or multi-layer fiber structures have used one in situ ALD process for all layers [16,26,27]. This makes the process less expensive, but at the same time, difficulties exist in burning the polymer without affecting the subsequent layer’s quality [26]. The improvement of the quality of hollow coaxial fiber structures by the proposed novel approach can be considered in several directions. In the annealing process, the more layers are deposited, the slower the process becomes. During the out-diffusion of the polymer, the crystallites of the upper films can be disoriented. Moreover, because of the different diffusion coefficients in adjacent metal oxide films, the Kirkendall effect takes place. During the annealing of ZnO/ALO/PVA fibers, besides the degradation and out-diffusion of PVA, vacancy diffusion starts from ALO to ZnO due to the different diffusion rates. This could result in a significant disruption of the microtubular morphology. Additionally, in the standard process for manufacturing hollow coaxial fiber structures, all the desired layers of the structure are deposited at a low temperature. This low temperature is necessary to protect the polymer from degradation and is usually well below the ALD temperature window for the respective material. This leads to the deposition of layers of poor quality. The method proposed in this work avoids the problems related to the annealing of multilayer fibers and also ensures the deposition of each layer within its own ALD window. Consequently, a significant improvement of the obtained structures could be attained. Therefore, this process, albeit more complicated, offers definite advantages as it allows the preparation of fibers with a better quality that is not deteriorated by the high-temperature annealing used to burn the polymer core.

## 4. Conclusions

The combination of electrospinning and atomic layer deposition is a powerful technique for the synthesis of HAR structures. We successfully obtained submicron ZnO (170 nm)/ALO (23 nm) hollow fibers with an inner cavity diameter of 512 nm on glass substrates. In our ex-situ ALD process, the burning and out-diffusion of polymers occurs only through the amorphous ALO film. We can then deposit the top ZnO films at high enough temperatures in order to obtain the desired preferential crystalline orientation. By using this sequence of technological processes, it becomes possible to prevent the Kirkendall effect and the significant deterioration and even destruction of the fibers that could occur during the high-temperature process to burn the polymeric core. This technology, although more complicated than the ones suggested previously, resulted in hollow fibers with a better quality. The results reveal that thin ZnO films deposited on the prepared ALO fibers have better crystallinity and fewer defects compared to the films deposited on flat Si surfaces under the same technological conditions. Therefore, in the novel approach proposed, unwanted effects are minimized and neutralized, and structures of a better quality are obtained. In addition, this approach could be applied to a wide range of materials, which otherwise could not withstand high-temperature processing. The obtained layered HAR structures of solid and hollow fibers, depending on the materials used, have enormous potential in various applications, such as nano- and biosensors, photonics and electronics, tissue engineering, filtering, catalysis, energy, supply of medicines, agriculture and the food industry, as smart textiles, and many others.

## Data Availability

The datasets that support the findings in this study are available from the corresponding author upon reasonable request.
